# Supplementary material for: Phenotype-based cell-specific metabolic modeling reveals metabolic liabilities of cancer
Source: eLife. 2014 Nov 21;3:e03641. doi: 10.7554/eLife.03641 (PMC4238051; doi:10.7554/eLife.03641)
Supplement: Supplementary file 1. — Supplementary files 1A–K. DOI: http://dx.doi.org/10.7554/eLife.03641.018 [file elife03641s001.docx]

**Supplementary Material**

**Supplementary Tables**

- Supplementary File 1A - Correlation results obtained by the E-Flux method
- Supplementary File 1B – Population composition of the HapMap dataset
- Supplementary File 1C – Pearson correlation between predicted and measured growth rate
- Supplementary File 1D – Drug response analysis for the HapMap dataset
- Supplementary File 1E - Drug response analysis for the CEU dataset
- Supplementary File 1F – Drug response analysis for the NCI-60 dataset
- Supplementary File 1G - Enrichment analysis of currently used cytostatic drugs
- Supplementary File 1H – MLYCD flux analysis
- Supplementary File 1I – Kaplan-Meier and COX regression analysis for PRIME model
- Supplementary File 1J – Kaplan-Meier analysis based on regression coefficients
- Supplementary File 1K – Kaplan-Meier analysis for iMAT and E-Flux

**Personalized metabolic modeling of lymphoblasts and cancer cell-lines**

|  | HapMap | | NCI-60 | |
| --- | --- | --- | --- | --- |
|  | Spearman Correlation | P-value | Spearman Correlation | P-value |
| Expression value | 0.11 | 0.07 | 0.43 | 4.8e-4 |
| Sigmoidal | 0.1 | 0.13 | 0.43 | 4.5e-4 |
| Exponential | 0.11 | 0.08 | 0.44 | 3.6e-4 |
| Polynomial | 0.1 | 0.1 | 0.44 | 4.1e-4 |

**Supplementary File 1A:** Correlation results obtained by the E-Flux[[1](#_ENREF_1)] method in both the Hap-Map and NCI-60 dataset using different functions.

| Populations | Number of samples |
| --- | --- |
| Utah residents with Northern and Western European ancestry | 56 |
| Han Chinese in Beijing, China | 43 |
| Japanese in Tokyo, Japan | 43 |
| Yoruba from Ibadan, Nigeria | 82 |

**Supplementary File 1B:** Population composition of the HapMap dataset

|  | Pearson rho | P-value |
| --- | --- | --- |
| HapMap | 0.44 | 3.8e-12 |
| NCI-60 | 0.68 | 1.18e-9 |

**Supplementary File 1C:** Pearson correlation between measured and predicted growth rate data

**Predicting individual cell-lines' drug response**

| Activation threshold | 5-fluorouracil (5FU) | | | | 6-mercaptopurine (6MP) | | | |
| --- | --- | --- | --- | --- | --- | --- | --- | --- |
|  | **Spearman Correlation** | **P-value** | **Spearman Partial Correlation** | **P-value** | **Spearman Correlation** | **P-value** | **Spearman Partial Correlation** | **P-value** |
| 10% | 0.19 | 0.0038 | 0.29 | 1.42e-05 | 0.17 | 0.03 | 0.25 | 0.0002 |
| 20% | 0.24 | 3.75e-4 | 0.32 | 1.59e-06 | 0.18 | 0.0078 | 0.28 | 3.63e-05 |
| 30% | 0.26 | 1.17e-4 | 0.33 | 9.33e-07 | 0.21 | 0.0019 | 0.29 | 1.26e-05 |
| 40% | 0.27 | 6.98e-5 | 0.32 | 1.88e-06 | 0.23 | 6e-4 | 0.3 | 7.95e-06 |
| 50% | 0.28 | 7.63e-5 | 0.3 | 5.33e-06 | 0.26 | 1.36e-4 | 0.31 | 3.48e-06 |
| 60% | 0.27 | 4.82e-5 | 0.3 | 5.81e-06 | 0.26 | 7.52e-5 | 0.3 | 5.83e-06 |
| 70% | 0.25 | 2.06e-4 | 0.26 | 6.96e-05 | 0.28 | 2.56e-5 | 0.3 | 4.87e-06 |
| 80% | 0.16 | 0.021 | 0.17 | 0.0119 | 0.27 | 6.32e-5 | 0.29 | 2.49e-05 |

**Supplementary File 1D:** Drug response results (HapMap). Drug response data used in this dataset was given as ATP concentration levels [[2](#_ENREF_2)]. For each of the lymphoblast models and for each activation thresholds (see Methods) we sampled the solutions space and obtained 1000 different flux distributions based on [[3](#_ENREF_3)]. The results in the table below are the mean Spearman correlation between measured and predicted drug response obtained by utilizing each of the 1000 sampled flux distributions as the wild-type flux distribution. The partial correlation results (i.e., controlling for the measured growth rate) are also reported in table.

| Drug Name | Spearman Correlation | P-value | Spearman partial Correlation | P-value |
| --- | --- | --- | --- | --- |
| Ethacrynic acid | 0.24 | 0.05 | 0.26 | 0.04 |
| Hexachlorophene | 0.24 | 0.05 | 0.25 | 0.04 |
| Digoxin | 0.26 | 0.05 | 0.26 | 0.05 |
| Azathioprine | 0.49 | 4e-4 | 0.49 | 4e-4 |
| Reserpine | 0.69 | 4e-5 | 0.69 | 4e-5 |

**Supplementary File 1E:** Drug response results (CEU). Drug response data used in this dataset was given as AC50 values [[4](#_ENREF_4)]. AC50 values represent the concentration in which the drug exhibits 50% of its maximum efficacy. In this case, *in silico* AC50 values are calculated by estimating the maximal flux rate carried by the target reaction when the growth rate is set to 50% of the drug's maximal response (a value that was available in the dataset used [[4](#_ENREF_4)]). The partial correlation results (i.e., controlling for the measured growth rate) are also reported in table. In cases where multiple reactions are associated with the drug's targets, the analysis is done for each reaction separately and the reported correlation is the mean over the correlation obtained for each reaction alone. In most cases similar correlations were obtained for different reactions targeted by the same drug. Two exceptions were found for the drugs Ethacrynic acid and Hexachlorophene in which the significant correlation of 0.24 was obtained for only one of the target reactions.

| Drug Name | Spearman Correlation | P-value |
| --- | --- | --- |
| Gemcitabine[[5](#_ENREF_5)] | 0.38 | 0.03 |
| Methotrexate[[5](#_ENREF_5)] | 0.4 | 0.02 |
| Trimetrexate[[6](#_ENREF_6)] | 0.45 | 2.3e-4 |
| Methotrexate[[7](#_ENREF_7)] | 0.47 | 1.18e-4 |
| Quinacrine HCl[[7](#_ENREF_7)] | 0.3 | 0.01 |

**Supplementary File 1F:** Drug response results (NCI-60). Drug response data used in this dataset was given as IC50 values [[5-7](#_ENREF_5)]. IC50 values represent the concentration of drug needed in order to reduce the growth to 50% of its maximal value. In this case, *in silico* IC50 values are calculated by estimating the maximal flux rate carried by the target reaction when growth rate is set to 50% of its maximal value. The partial correlation results (i.e., controlling for the measured growth rate) in this case were insignificant (P-value > 0.05).

**Predicting cancer drug targets with a selective effect on cell proliferation**

| Threshold (% of reduction in maximal growth rate) | Hypergeometric Enrichment P-value | Hypergeometric enrichment (Cox regression analysis) |
| --- | --- | --- |
| 10% | 4.05e-7 | 0.77 |
| 20% | 4.24e-4 | 0.49 |
| 30% | 0.0031 | 0.88 |
| 40% | 1.09e-4 | 0.74 |
| 50% | 1.07e-5 | 0.33 |

**Supplementary File 1G:** The effect of reaction's deletion on cell proliferation for the identification of selective treatment was simulated as described in the Methods part. The overlap between the set of 24 cytostatic drug targets taken from Folger et al. [[8](#_ENREF_8)] (drugs classified as "Metabolic Anticancer Drug Targets"), and our predictions was found to be robust to different thresholds that determine the value (in percentage) under which the deletion is considered to effect the cell's proliferation rate. The table describes the enrichment found for different thresholds. The mean over these values is the one reported in the paper.

| Metabolic Pathway | Reaction Formula | One-sided Wilcoxon P-value |
| --- | --- | --- |
| Pentose Phosphate Pathway | g6p[c] + nadp[c] <=> 6pgl[c] + h[c] + nadph[c] | 1.28E-34 |
| Pentose Phosphate Pathway | 6pgc[c] + nadp[c] => co2[c] + nadph[c] + ru5p_DASH_D[c] | 1.28E-34 |
| Pentose Phosphate Pathway | 6pgl[c] + h2o[c] => 6pgc[c] + h[c] | 1.28E-34 |
| Glutamate metabolism | gthox[c] + h[c] + nadph[c] => 2gthrd[c] + nadp[c] | 2.82E-39 |
| Glutathione Metabolism | 2gthrd[c] + h2o2[c] <=> gthox[c] + 2h2o[c] | 6.08E-28 |
| Fatty acid elongation | 3h[c] + malcoa[c] + 2nadph[c] + pmtcoa[c] => co2[c] + coa[c] + h2o[c] + 2nadp[c] + stcoa[c] | 1.28E-34 |
| Fatty acid elongation | 3h[c] + malcoa[c] + 2nadph[c] + tdcoa[c] => co2[c] + coa[c] + h2o[c] + 2nadp[c] + pmtcoa[c] | 1.28E-34 |
| Fatty acid elongation | 3h[c] + malcoa[c] + 2nadph[c] + occoa[c] => co2[c] + coa[c] + dcacoa[c] + h2o[c] + 2nadp[c] | 1.28E-34 |
| Fatty acid elongation | dcacoa[c] + 3h[c] + malcoa[c] + 2nadph[c] => co2[c] + coa[c] + ddcacoa[c] + h2o[c] + 2nadp[c] | 1.28E-34 |
| Fatty acid elongation | ddcacoa[c] + 3h[c] + malcoa[c] + 2nadph[c] => co2[c] + coa[c] + h2o[c] + 2nadp[c] + tdcoa[c] | 1.28E-34 |
| Fatty acid elongation | accoa[c] + 9h[c] + 3malcoa[c] + 6nadph[c] => 3co2[c] + 3coa[c] + 3h2o[c] + 6nadp[c] + occoa[c] | 1.28E-34 |
| Fatty acid elongation | accoa[c] + 20h[c] + 7malcoa[c] + 14nadph[c] => 7co2[c] + 8coa[c] + 6h2o[c] + hdca[c] + 14nadp[c] | 2.82E-39 |

**Supplementary File 1H:** Flux analysis for MLYCD knockdown. Utilizing the RPMI-8226 model we first obtained 1000 wild-type flux distribution via Flux Balance Analysis (FBA), in which the MLYCD reaction is forced to be active in a rate that is at least 50% of the maximal flux rate it can carry. Next, the knockout flux distribution is computed via MOMA while constraining the flux through the corresponding reactions to zero. Utilizing the 1000 pre- and post-knockout flux distributions, we applied a one-sided Wilcoxon ranksum test to determine reactions whose flux has been significantly increased/decreased. The table summarizes relevant reactions whose flux has been significantly increased (red) or decreased (green). Specifically, we find an increase in the oxidative-branch of the pentose phosphate pathway that generates NADPH. This cofactor is necessary in order to meet the increasing demands of the fatty acid synthesis pathway. This in turn results with a decreased flux through the reaction that generates reduced glutathione and then utilizes it to detoxify ROS.

**Reconstructing personalized metabolic models based on clinical samples obtained from cancer patients**

|  | Miller et al. | Chang et al. | Okayama et al. |
| --- | --- | --- | --- |
| Number of patients | 236 | 295 | 226 |
| Kaplan-Meier (log-rank p-value) | 0.01 | 1e-3 | 0.02 |
| Cox univariate analysis | 1e-3 | 1.05e-4 | 2e-3 |
| Cox multivariate analysis | 0.019 | 5.9e-4 | 0.13 |

**Supplementary File 1I:** Summary of the Kaplan-Meier and Cox analyses for the predicted growth rate in the three clinical datasets analyzed in the main text. Clinical predictors of breast cancer survival that were available in each of the datasets are: (1)

Miller et al.: Age, tumor size, histological grade, lymph node status and estrogen receptor status; (2) Chang et al.: Age, tumor size and metastasis; (3) Okayama et al.: Age and tumor stage

|  | Miller et al. | Chang et al. | Okayama et al. |
| --- | --- | --- | --- |
| Kaplan-Meier (log-rank p-value) | 0.23 | 8.5e-4 | 0.65 |
| Empiric P-value 1 | 0.29 | 0.075 | 0.7 |
| Empiric P-value 2 | 0.3 | 0.067 | 0.69 |
| % of genes overlapping with the GR-associated genes according to the NCI-60 dataset | 6% | 7.3% | 8.9% |

**Supplementary File 1J:** To examine the potential added value of personalized metabolic models in predicting patients' prognosis, we repeated the survival analysis using the raw gene expression. Namely, we utilized the NCI-60 gene expression data while considering only the set of genes found in the human metabolic model, and applied multiple linear regression analysis to estimate the model's parameters. The latter were then used to predict the growth rates of the individual samples found in the three cohorts analyzed here. Supplementary table S10 summarizes the: (1) Kaplan-Meier log-rank p-value; (2) an empiric p-value computed by permuting the regression coefficients values 1000 times (Empiric P-value 1); (3) an empiric p-value computed by permuting the NCI-60 gene expression data 1000 times (Empiric P-value 2); (4) The overlap in percentage between the genes identified by the regression analysis, and the genes that are correlated to the cell-lines' growth rate according to the NCI-60 data (for a full list see Supplementary Dataset S1). The obtained results testify that personalized metabolic modeling bears an advantage in predicting patients' prognosis over using the gene expression alone in a model-free manner.

|  | Miller et al. | Chang et al. | Okayama et al. |
| --- | --- | --- | --- |
| E-Flux | 0.32-0.47 | --- | 0.57-0.93 |
| iMAT | 0.16 | 0.02* | 0.12 |

**Supplementary File 1K:** Summary of Kaplan-Meier log-rank p-values when applying either E-Flux or iMAT. The Chang et al. dataset includes negative values which is not clear how they should be considered in the E-Flux pipeline. We therefore chose not to run the E-Flux method on this dataset. *Additionally the significant results obtained by iMAT for this dataset indicate that *high growth rate* is associated with better survival which is more difficult to explain.

**References**

1. Colijn, C., et al., *Interpreting Expression Data with Metabolic Flux Models: Predicting Mycobacterium tuberculosis Mycolic Acid Production.* PLoS Computational Biology, 2009. **5**(8): p. e1000489.

2. Choy, E., et al., *Genetic Analysis of Human Traits In Vitro: Drug Response and Gene Expression in Lymphoblastoid Cell Lines.* PLoS Genetics, 2008. **4**(11): p. e1000287.

3. Bordel, S., R. Agren, and J. Nielsen, *Sampling the Solution Space in Genome-Scale Metabolic Networks Reveals Transcriptional Regulation in Key Enzymes.* PLoS Computational Biology, 2010. **6**(7): p. e1000859.

4. Lock, E.F., et al., *Quantitative High-Throughput Screening for Chemical Toxicity in a Population-Based In Vitro Model.* Toxicological Sciences, 2012. **126**(2): p. 578-588.

5. Garnett, M.J., et al., *Systematic identification of genomic markers of drug sensitivity in cancer cells.* Nature, 2012. **483**(7391): p. 570-575.

6. Scherf, U., et al., *A gene expression database for the molecular pharmacology of cancer.* Nature Genetics, 2000. **24**(3): p. 236-244.

7. Holbeck, S.L., J.M. Collins, and J.H. Doroshow, *Analysis of Food and Drug Administrationג€“Approved Anticancer Agents in the NCI60 Panel of Human Tumor Cell Lines.* Molecular Cancer Therapeutics, 2010. **9**(5): p. 1451-1460.

8. Folger, O., et al., *Predicting selective drug targets in cancer through metabolic networks.* Molecular Systems Biology, 2011. **7**: p. 501.
